# Coloured Filters Enhance the Visual Perception of Social Cues in Children with Autism Spectrum Disorders

**DOI:** 10.5402/2012/298098

**Published:** 2012-03-04

**Authors:** Amanda K. Ludlow, Elaine Taylor-Whiffen, Arnold J. Wilkins

**Affiliations:** ^1^Department of Psychology, University of Birmingham, Edgbaston, Birmingham B15 2TT, UK; ^2^Department of Psychology, Anglia Ruskin University, East Road, Cambridge CB1 1PT, UK; ^3^Department of Psychology, University of Essex, Colchester, Essex CO4 3SQ, UK

## Abstract

Coloured filters have been found to reduce visual distortion of text in children with autism spectrum disorders (ASD). We investigated the effect of the overlays on the “mind in the eye” task in children with ASD and controls matched for age, gender, and nonverbal IQ. Children were shown photographs of the periocular region of various faces and were asked to judge which emotion was being expressed in the eyes. In children with ASD, the perception of the emotion was significantly improved when the photograph was covered by a coloured overlay. The improvement was significantly greater than in the controls, who showed no significant effect of the overlay. A perceptual impairment may contribute to the social difficulties shown in ASD.

## 1. Introduction

There is a growing body of evidence that unusual sensory processing is associated with autism spectral disorders (ASD) and may underlie some of their basic characteristics. To date, there is relatively little systematic evidence as to the pattern of atypical sensory behaviours in autism and how they relate to other characteristics of the disorder. In this paper, visual disturbance will be considered and it will be shown that it may be responsible for high levels of social dysfunction.

Children with ASD also present a range of deficits in processing information that appears social in nature. Importantly, these social deficits appear to have a visual component. For example, infants with ASD tend to be less attentive to people and many other social cues in their environment [1–3]. They make less frequent and abnormally timed eye contact [4, 5]. They have also been reported to show poor orientation to and discrimination of faces and poorer recognition of emotional expressions [3, 6–12]. It is possible that a basic perceptual difficulty might play a significant if not primary contributory role in the difficulties children with ASD have in processing facial stimuli [11, 13].

Standardised parental questionnaires have shown that as many as 95 percent of individuals with ASD present high frequencies of “sensory behaviours”. Whole body, hand and finger mannerisms, and unusual sensory interests (especially visual objects) have also been shown to discriminate children with autism from those without [14]. These “sensory behaviours” suggest the possibility of a heightened sensory sensitivity reminiscent of that characterised in other literature as visual stress [15, 16] and treated using coloured filters.

Visual stress refers to visual discomfort from exposure to disturbing visual patterns and often is cited in relation to reading difficulties, light sensitivity, and headaches. Visual stress is thought to be responsible for print distortion and may contribute to rapid fatigue when reading [16]. Anecdotal reports frequently suggest that to individuals with autism, the world seems disjointed, confused, and scary (e.g., [17]). For example, other people and things may seem blurry, move around, or even disappear. These odd sensory perceptions evidenced in ASD appear to resemble “visual stress.”

A large proportion of individuals with ASD show perceptual benefits from the use of coloured filters [18–20]. The overlays are designed to sample chromaticity systematically and comprehensively so that if there is any colour that is beneficial, there is available an overlay or combination of overlays providing a close approximation to this colour [21]. Children (both those with ASD and those without) who benefit from the filters report distortions of the text and discomfort when reading, and read more quickly with the provision of a coloured filter. The proportion of those with ASD that benefit in this way is four times higher than in the general population [18]. In one individual, J. G. [20], the wearing of tinted spectacles resulted in remarkable beneficial changes in behaviour, such as a greater awareness of both his and other people's personal space and improvements in social function. Thus, coloured filters have also been found to aid both visuospatial ability and social function [19, 20]. Importantly, it has been shown the effects of these overlays are not readily attributable to demand characteristics and that these perceptual benefits extend to visual search and matching to sample [19].

Whiting and Robinson [22] were the first to show in groups of children suffering from visual stress significant impairment in the ability to correctly interpret facial emotion. Importantly, this was found not to relate to any level of learning disability [23]. In both studies, children who suffered from visual stress showed better facial recognition when using a coloured overlay.

The mind in the eye task [24] involves looking at photographs of the eye region of faces, and choosing which of four words best describes how the person in the photograph is feeling. It is often seen as a subtle theory-of-mind task because it incorporates mental states and is therefore more than just a test of emotion perception [25]. We examined the effect of coloured filters on the performance of the mind in the eye task in children with autism and age- and sex-matched controls.

## 2. Method

### 2.1. Participants

15 children (3 female, 12 male) with a diagnosis of autism spectrum disorders (ASD) participated in the study, 11 children had a formal diagnosis of autism and 4 had a diagnosis of Asperger Syndrome. They were aged between 8 years 2 months and 17 years 2 months (mean 13 years) and were recruited from two schools for children with moderate learning disabilities with autism units and a mainstream school. Control participants were sampled from two mainstream schools. These children were matched individually to the children with ASD for chronological age, gender, and scores on Ravens Matrices [26] (nonverbal IQ). All children were native monolingual speakers of English. Their parents confirmed that participating children had not and did not suffer from any hearing problems or psychopathological or neurological disorders. The children's psychometric data are shown in Table 1.

The autism diagnostic observation schedule generic (ADOS-G) [27] was carried out on the ASD group to confirm their diagnosis of autism and to gain additional information about their social and language behaviour. The protocol consists of a series of structured and semi-structured tasks that involve social interaction between the examiner and the participant. All ASD participants had an unambiguous clinical diagnosis of autistic disorder or Asperger's syndrome according to DSM-IV criteria and scored above threshold for ASD on the ADOS-G diagnostic algorithm. None had identifiable medical conditions underlying their ASD.

### 2.2. Materials

The *rate of reading test* [28] and the *intuitive overlays* [21] were used in the study. The Reading test consists of paragraphs of unrelated words. It is published in two versions that differ in typeface, size, and spacing. In this study, the larger text (14pt Geneva) was used.

The *Children's version of “reading the mind in the eyes” test *[24] consists of 28 pictures of the eye region of the face. The subject is asked to pick which of 4 words surrounding the photograph best describes what the person in the photograph is thinking or feeling. The test is the result of piloting with typically developing children. Three of the 4 words are foil mental state terms, and the other word is “correct”. The position of the four words is randomised. The item analysis reported by Baron-Cohen et al. shows that the difficulty of the items did not vary systematically with serial position (the correlation between the serial position of an item and the probability of a correct response was 0.05). The test was therefore divided into two halves of equal difficulty, one consisting of items 1–14, and the other items 15–28.

*The Intuitive Overlays* [21] were used in the study. These are coloured plastic sheets. They are supplied in a teacher's pack and include two A5 size overlays of each of the following colours, rose, orange, yellow, lime green, mint green, aqua, blue, purple, pink, and grey. The spectral reflectances were given by Wilkins [21].

### 2.3. Procedure

Full ethical approval was granted to complete these studies and all children and their parents' gave their informed consent prior to their/their child's inclusion in the study.

In order to familiarise the children with the *rate of reading test*, they were presented with a short passage from the test and asked to read out loud for 30 seconds. The children were then presented with two identical passages from the *rate of reading test* side by side to compare when assessing which overlay made the text clearer. The passages had 20 lines of 15 words and were printed in Arial 10pt with 8pt word spacing and 1.0 line spacing. The preferred coloured overlay was chosen according to the procedure recommended in the manual.

### 2.4. Rate of Reading

One of the passages of text was then removed. The other passage was then read with or without an overlay placed upon the page, in an order that was balanced across subjects, selected at random. The children were asked to read the text aloud for one minute as quickly as possible and were timed using a stopwatch. The total number of words read in the correct order was noted.

### 2.5. Children's Mind in the Eye Task

Children were told they were to see pictures of people's eyes each with four words around them. The children were to look carefully at the picture and then choose the word that best describes what the person in the picture is thinking or feeling. For example during the practice trial they were shown a person and asked “Do you think he is feeling jealous, scared, relaxed, or hated?” The words were pointed to as they were read out. All children were required to pick one and encouragement was given to each child whether they were right or wrong. After the practice trial, children proceeded to main trials which were carried out in the same way. Children were told to guess when they did not know.

The 28 pictures were divided into two series comprising the first and the second 14 items. Half of the participants experienced items 1–14 first and half items 15–28 first. Half the participants in each of these groups, chosen at random, undertook the first series with an overlay, the second series without, and the other half used the reverse orders.

## 3. Results

An analysis of variance was carried out between groups to confirm that no differences existed between groups in terms of matching criteria. There were found to be no significant differences in nonverbal ability *F*(1,29) = 1.79, *P* = .19 and age of the children *F*(1,29) = .65, *P* = .43.

### 3.1. Rate of Reading

The rate of reading with and without an overlay is shown for both groups in Table 2.

In order to examine the difference in rate of reading with and without an overlay, a 2∗2 mixed analysis of variance was carried out between groups. This revealed a significant effect of condition, *F*(1,28) = 15.89, *P* = .001, more words being read with an overlay (mean = 108.2; sd = 35.9) than without (mean = 101.9; sd = 34.9), *t*(29) = 3.85, *P* < 0.001. There was a significant effect of group, *F*(1,28) = 5.53, *P* < .05. The control group read significantly faster without an overlay compared to the autistic group, *t*(28) = 2.02, *P* < .05, (mean = 117.3 (29.7) and 86.5 (33.7), resp.). However, there were no significant differences between the control and the autism group as regards the number of words read with an overlay, *t*(28) = .54, *P* = .06 (mean = 120.8 (30.6) and 95.6 (37.2), resp.).

There was a marginal group by condition interaction, *F*(1,28) = 3.11, *P* = .08, and further inspection of the data revealed the children with autism showed significantly better performance with an overlay *t*(14) = 3.77, *P* < .005, whereas the controls failed to show significantly better performance with an overlay *t*(14) = 1.72, *P* = .11, see Table 2.

In previous studies [29, 30], the criterion for clinically significant improvements in reading speed when using overlays has been set at 5%. Substantially greater numbers of children with autism 12/15 (80%) read > 5% faster with an overlay, with levels of improvement of up to 39%. In comparison, only 5/15 (33%) of the typically developing children read more than 5% faster with an overlay. Therefore significantly more autistic children than controls reached the level of improvement at which overlays are considered to be an advantage, *X* ^2^(1) = 6.7, *P* < .05.

There was no significant difference between groups in the number of symptoms of visual stress reported with an overlay *F*(1,29) = .12, *P* = .72 and without an overlay *F*(1,29) = 2.25, *P* = .15. For both groups, the number of symptoms with the overlay was less than without, although this failed to reach significance, *t*(29) = 1.65, *P* = .11.

### 3.2. Reading the Mind in the Eye Task

The principal aim of the current study was to assess whether overlays could lead to an improvement on the reading the *mind in the eye* task. In order to examine the difference in performance with and without an overlay, a two-way analysis of variance of errors was carried out between groups. This revealed no significant effect of condition, *F*(1,28) = , 71, *P* = .40. However, there was a significant effect of group, *F*(1,28) = 20.99, *P* < .001 and a significant group ∗ condition interaction, *F*(1,28) = 5.53, *P* < .05.

The autism group were found to be significantly poorer than the controls at the number of emotions identified both without an overlay *t*(28) = 5.14, *P* < .0001 and with *t*(28) = 3.11, *P* < .005. Within groups, the children with autism showed significantly better performance with an overlay *t*(14) = 2.37, *P* < .05, *η* ^2^ = .16, identifying more emotions correctly with an overlay, mean 7.1 (2.4) than without 6.0 (2.6). Whereas the controls failed to show a significant difference in performance with an overlay *t*(14) = .12, *P* = .33, *η* ^2^ = .04, identifying a similar number of emotions with an overlay, mean = 10.2 (1.7) as without 9.7 (2.1). The mean performance for both groups across conditions is shown in Table 2.

11/15 (73%) of the children with autism identified more faces with an overlay. In comparison, only 4/15 (27%) of controls identified more faces correctly, significantly fewer, *X* ^2^(1) = 6.53, *P* < .05.

**Figure 1:**
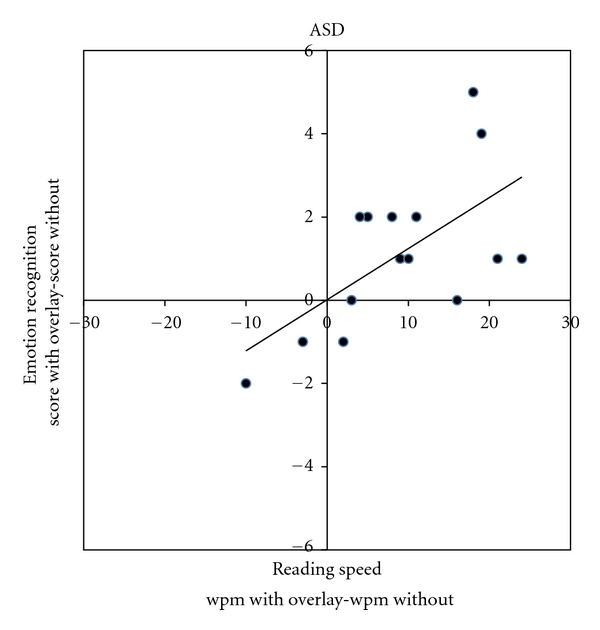
Differences in level of performance with an overlay for each participant for the rate of reading and mind in the eye tasks.

**Figure 2:**
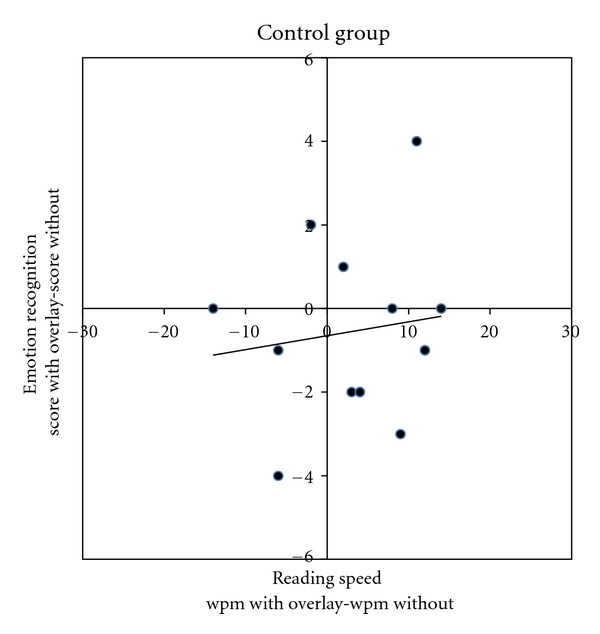
Differences in level of performance without an overlay for each participant for the rate of reading and mind in the eye tasks.

Figures 1 and 2 demonstrate that the children with autism who read faster with an overlay also correctly identified more emotions with the use of an overlay *r*(15) = 0.57, *P* < 0.02. The association is weaker *r*(15) = 0.14 and nonsignificant for the controls. The number of emotions identified correctly was not related to age in either group (ASD Group, *r*(15) = −0.16, *P* = .56); controls, *r*(15) = 0.064, *P* = .82) nor nonverbal ability (ASD Group, *r*(15) = −0.006, *P* = .98; controls, *r*(15) = −0.31, *P* = .26).

## 4. Discussion

There is a growing consensus that perceptual alterations may well be a core characteristic of autism [31]. The current findings are important in showing that perceptual abnormalities in a large proportion of children with autism benefit from the use of an overlay, not just in respect of reading ability, but also as regards improvements in perception of facial expression of emotions. This then provides further evidence that low-level perceptual abnormalities may be responsible for their difficulties attending and processing facial expressions [11].

The current study revealed 80% of children improved reading with the use of a coloured filter and this is consistent with previous studies [18, 19]. Thus, the majority of individuals with autism appear to show beneficial effects with the use of coloured filters. Whilst these perceptual benefits have also been shown to extend to both visual search and matching to sample tasks [19], this is the first study to show that a similar proportion of children with autism may also benefit on tasks of social perception such as “the mind in the eye task.”

The results replicate previous studies in showing that children with ASD were significantly poorer at identifying emotions on the “mind in the eye task” compared to controls [24]. Facial expression of emotions has profound implications for social understanding [32] and deficits in social perception are among the core characteristics needed for a diagnosis of autism. Little is known about the underlying reasons for such deficits but the current findings suggest that a perceptual impairment may contribute. For example, the inability to meet or respond to social signals expressed in the eyes [33] may lead to a failure to engage in social intercourse, a precursor to deficits in theory of mind [34].

Aside from autism, precision spectral filters have been shown to offer symptom relief in several central nervous system disorders that involve the visual system, including photosensitive epilepsy [35], multiple sclerosis [36], and migraine [37]. These disorders are associated with an increased risk of seizures, consistent with the hypothesis that coloured filters reduce the effects of a cortical hyperexcitability. Cortical hyperexcitability is likely in ASD given the comorbidity with epilepsy [38]. Indeed a number of authors have suggested that neural noise may be increased in individuals with ASD or that increased levels of noise may contribute to poorer performance on cognitive tasks in those with ASD [39, 40]. In particular, some have suggested that cortical hyperexcitation especially in primary sensory cortices would lead to increased cortical noise in ASD [39]. Coloured filters have been shown to reduce the effects of increased neural noise in dyslexic individuals [41].

## Figures and Tables

**Table 1:** Mean (and standard deviation) of age, and scores on the Ravens Progressive Matrices for 15 children with ASD and their 15 controls.

Group	Age	Ravens
ASD (*N* = 15)	13.0 (6.2)	84.7 (17.4)
Controls (*N* = 15)	12.4 (4.1)	94.9 (21.8)

**Table 2:** Means and SD for performance on the rate of reading tasks and reading the mind in the eye for the group with ASD and the control group.

	Number of words per minute		Number of emotions identified	
	Without	Overlay	Without	Overlay
ASD (*N* = 15)	86.5 (33.7)	95.6 (37.2)	6.0 (2.6)	7.1 (2.4)
Controls (*N* = 15)	117.3 (29.7)	120.8 (30.6)	10.2 (1.7)	9.7 (2.1)
